# 12-month weight-loss trajectories and predictors of response to tirzepatide under individualized dosing: A multicenter real-world study

**DOI:** 10.1016/j.obpill.2026.100344

**Published:** 2026-10-12

**Authors:** Nikolaos Angelopoulos, Evangelos Fousteris, Dimitra Zianni, Ioannis Androulakis, Rodis Paparodis, Dimos Florakis, Andreas Rizoulis, Ilias Perogamvros, Ploutarchos Tzoulis, Dimitrios Askitis, Georgia Argyrakopoulou, Evdoxia Gitsi, Sarantis Livadas, Zadalla Mouslech, Valentina Petkova, Alexander Kokkinos, Vaia Lambadiari

**Affiliations:** a Hellenic Endocrine Network, Ermou 6, Athens, Greece; b Mediterranean Diabetes and Obesity Clinics (MEDOC), Athens, Greece; c Department of Endocrinology, Metropolitan General Hospital, Athens, Greece; d Division of Diabetes, Endocrinology and Gastroenterology, School of Medical Sciences, University of Manchester, UK; e Division of Medicine, Department of Metabolism & Experimental Therapeutics, University College London Medical School, London, UK; f Diabetes and Obesity Unit, Athens Medical Center, Athens, 15125, Greece; g 1st Medical Propedeutic Department of Internal Medicine, AHEPA University Hospital, Aristotle University of Thessaloniki, Thessaloniki, Greece; h 1st Department of Propaedeutic Internal Medicine, Medical School, National and Kapodistrian University of Athens, Laiko General Hospital, Athens, Greece; i 2nd Department of Internal Medicine, Research Institute and Diabetes Center, National and Kapodistrian University of Athens Medical School, Athens, Greece

**Keywords:** Tirzepatide, Obesity, Real-world evidence, Weight loss, Treatment persistence

## Abstract

**Background and aims:**

Real-world evidence on 12-month treatment effectiveness and persistence with tirzepatide remains limited, particularly under individualized dosing and self-funded treatment conditions. We evaluated 12-month tirzepatide outcomes in routine clinical practice.

**Methods:**

This was a multicenter, retrospective, observational real-world study including 249 adults with overweight or obesity treated with tirzepatide across 13 obesity practices in Greece. Treatment was entirely self-funded, and dose escalation was individualized rather than protocol driven. Treatment persistence, reasons for discontinuation, weight loss, dose trajectories, metabolic outcomes, and predictors of 12-month response were evaluated.

**Results:**

At 12 months, 170/249 patients (68.3%) remained on treatment, 64 (25.7%) had confirmed discontinuation, and 15 (6.0%) were lost to follow-up. Among patients with available weight measurements, mean weight loss was 19.9 ± 8.6% at 12 months, with 88.9%, 50.0%, and 27.2% achieving ≥10%, ≥20%, and ≥25% weight loss, respectively. Mean tirzepatide dose at 12 months was 9.71 mg. Among 179 patients assessed at both 6 and 12 months, 70.4% experienced >2% additional weight loss during the second 6 months, whereas 10.1% experienced >2% weight regain. Six-month percentage weight loss was not significantly associated with additional weight loss between months 6 and 12 after multivariable adjustment (β = −0.110, P = 0.135). Among 139 participants who had not achieved ≥20% weight loss at month 6, early response demonstrated moderate discrimination for achieving this cumulative threshold at month 12 (AUC 0.760, 95% bootstrap CI 0.675–0.837), with an adjusted OR of 1.34 per additional percentage point of 6-month weight loss (P < 0.001).

**Conclusions:**

In a fully self-funded real-world setting, tirzepatide was associated with substantial weight loss and high documented treatment persistence over 12 months. Six-month weight-loss response was associated with the likelihood of achieving ≥20% weight loss at 12 months. These findings do not establish long-term weight maintenance beyond the observation period.

## Graphical abstract

**Figure:**
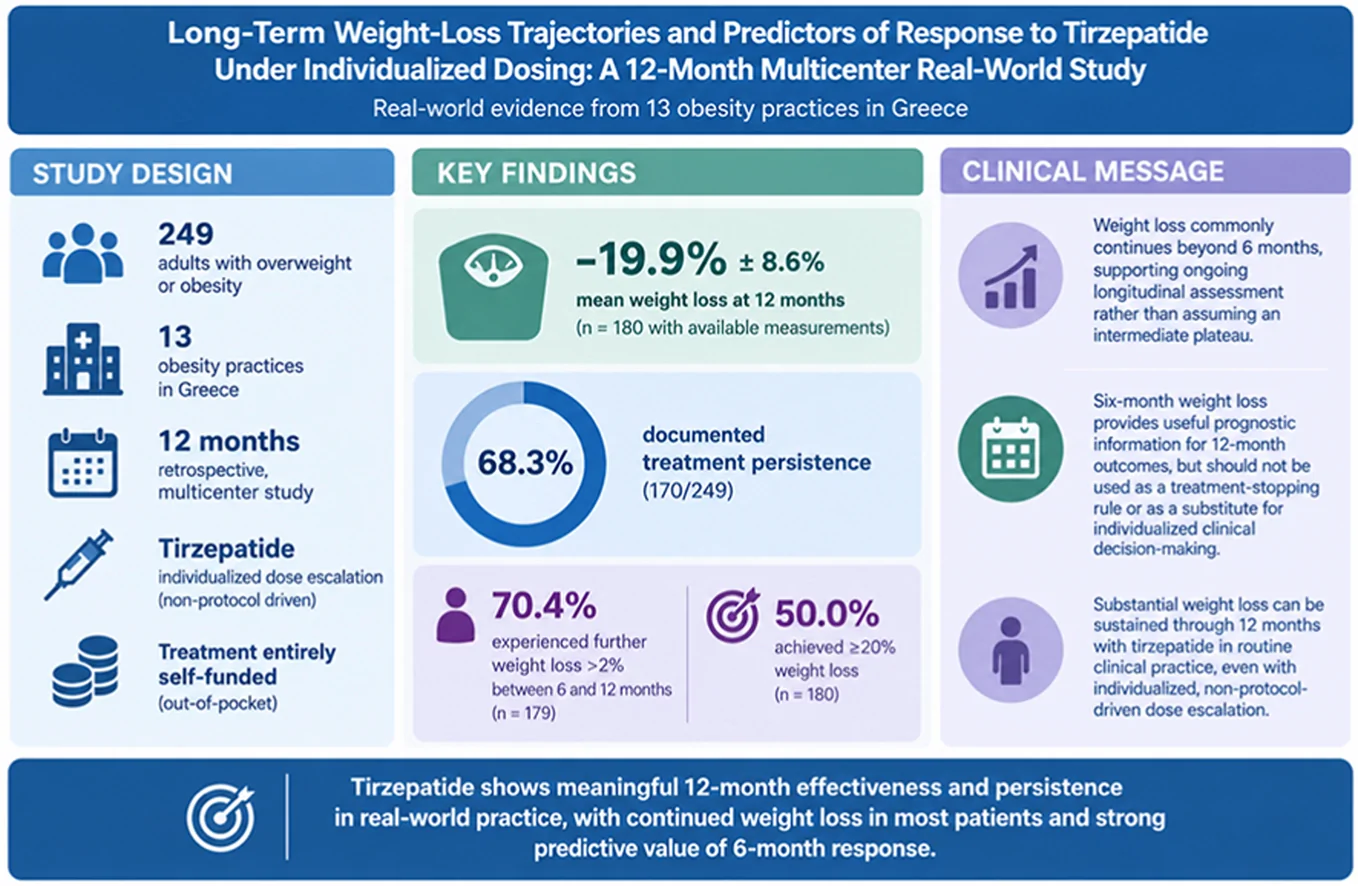

## 1 Introduction

Obesity is a chronic, relapsing disease that frequently requires sustained treatment to achieve and maintain clinically meaningful weight loss [1]**.** The advent of incretin-based pharmacotherapy has substantially changed its therapeutic landscape, with tirzepatide, a dual glucose-dependent insulinotropic polypeptide (GIP) and glucagon-like peptide-1 (GLP-1) receptor agonist, producing marked and sustained reductions in body weight together with broad cardiometabolic benefits [2]**.** In SURMOUNT-1, mean weight reduction at 72 weeks ranged from 15.0% with tirzepatide 5 mg to 20.9% with 15 mg under the treatment-regimen estimand, establishing the substantial efficacy of tirzepatide for obesity treatment [3]**.** However, outcomes achieved under randomized trial conditions may not fully translate into routine clinical practice, where treatment exposure, dose escalation, follow-up intensity, tolerability, patient preferences, and access to therapy are considerably more heterogeneous [4]**.**

Growing real-world evidence supports the effectiveness of tirzepatide outside randomized trials [5], [6]**.** Large database studies have demonstrated clinically meaningful weight loss despite slower dose escalation and lower treatment persistence than typically observed in clinical trials [4], [7]**.** Similarly, an academic obesity-clinic cohort showed that real-world titration and persistence were variable and that patients remaining on GLP-1–based therapy for at least 12 months achieved substantial weight loss [8]**.** Our group has also previously reported significant short-term weight loss and metabolic improvement with tirzepatide in Greek real-world cohorts [9], [10], [11]**.** Nevertheless, clinically important questions remain regarding 12-month treatment persistence, reasons for discontinuation, individualized dose trajectories, and the extent to which weight loss continues beyond the first 6 months of treatment.

Early treatment response may also provide clinically useful information regarding subsequent weight-loss outcomes [9], [12]**.** A post hoc analysis of SURMOUNT-1 demonstrated that greater early weight reduction was associated with greater subsequent weight loss, while many individuals classified as early poor responders nevertheless achieved clinically meaningful weight reduction with continued treatment [13]**.** Whether intermediate weight loss can similarly identify patients likely to achieve substantial 12-month weight reduction under routine-care conditions remains less well established. This question is particularly relevant in real-world practice, where treatment intensity is adapted to individual response rather than determined solely by a standardized titration protocol.

Treatment persistence may be especially challenging in healthcare settings in which access to obesity medications depends directly on patients' ability and willingness to sustain its cost [14]**.** During the period covered by the present study, tirzepatide prescribed for obesity in Greece was not reimbursed and treatment was paid entirely out of pocket. Thus, continuation and dose-related decisions reflected not only effectiveness and tolerability but also individual treatment goals, patient preferences, and affordability. This setting provides an opportunity to examine 12-month tirzepatide treatment patterns and outcomes under conditions that differ meaningfully from both randomized trials and real-world cohorts in which medication costs are fully or partly covered.

Against this background, we conducted a multicenter retrospective real-world study to characterize tirzepatide treatment through 12 months among adults with overweight or obesity treated in routine clinical practice in Greece. We evaluated treatment persistence and reasons for discontinuation, weight-loss effectiveness and metabolic outcomes, and real-world dose trajectories. We additionally characterized weight-change patterns between 6 and 12 months and examined whether 6-month weight-loss response predicted achievement of ≥20% weight loss at 12 months.

## 2 Materials and methods

### 2.1 Study design and population

This was a multicenter, retrospective, observational real-world study evaluating 12-month treatment persistence, effectiveness, treatment patterns, and metabolic outcomes among adults receiving tirzepatide for overweight or obesity in routine clinical practice in Greece. Data were retrospectively extracted from routine clinical records across 13 participating obesity practices. All consecutive eligible patients who initiated tirzepatide between January 1 and July 31, 2025, were included, irrespective of subsequent treatment continuation or the availability of follow-up weight measurements. The database cutoff date was July 31, 2026, ensuring that all included patients had the opportunity to complete 12 months of follow-up after treatment initiation. Patients who discontinued treatment or were lost to follow-up remained part of the baseline cohort. Eligible patients were adults aged ≥18 years who had initiated tirzepatide for weight management and had a body mass index (BMI) ≥30 kg/m^2^, or a BMI >27 kg/m^2^ in the presence of at least one weight-related comorbidity. Patients with advanced chronic kidney disease (stage 4 or 5; estimated glomerular filtration rate [eGFR] <30 mL/min/1.73 m^2^), active liver disease, or a personal or family history of medullary thyroid carcinoma were excluded.

Tirzepatide had been prescribed as part of routine clinical care, independently of the present retrospective analysis. Treatment decisions were made by the treating physician in consultation with the patient and were not determined by a study protocol.

Importantly, throughout the entire observation period, tirzepatide prescribed for obesity was not reimbursed in Greece, and treatment costs were borne entirely by patients. Accordingly, treatment initiation, continuation, dose adjustment, and discontinuation occurred in a fully self-funded, out-of-pocket setting.

### 2.2 Tirzepatide treatment and follow-up

Tirzepatide was administered once weekly by subcutaneous injection. All patients initiated treatment at a dose of 2.5 mg once weekly. Subsequent dose escalation was not mandatory and was not governed by a protocol-defined titration schedule. Dose adjustments were individualized according to treatment response, tolerability, patient preference, affordability, and the clinical judgment of the treating physician. When treatment intensification was considered appropriate, the dose could be increased in 2.5-mg increments within the approved dose range.

Decisions regarding dose escalation, maintenance, dose reduction, or treatment discontinuation reflected routine clinical practice. No study-mandated target dose, weight-loss target, or treatment algorithm determined subsequent management. Lifestyle management was likewise not standardized by the study; patients received routine advice regarding dietary modification and physical activity according to usual clinical practice.

Patients receiving tirzepatide were monitored approximately monthly through in-person visits or telephone consultations, during which ongoing treatment use, temporary interruptions, and discontinuations were assessed and documented in the clinical records. For the present analysis, treatment persistence was retrospectively evaluated using these records. Temporary treatment interruptions of up to two consecutive missed weekly injections (approximately 15 days) were permitted, provided that treatment was subsequently resumed. Interruptions exceeding 15 days were classified as treatment discontinuation, even if tirzepatide was restarted at a later time. At approximately 6 and 12 months after treatment initiation, patients were classified as continuing tirzepatide, having confirmed treatment discontinuation, or being lost to follow-up. Patients whose treatment status could not be reliably established were classified as lost to follow-up and were not assumed to have discontinued treatment.

For patients with confirmed treatment discontinuation, the documented reason was extracted from the clinical record and categorized as adverse events, achievement of the individualized treatment goal, treatment cost, or inadequate treatment response. Discontinuations were additionally classified as occurring within the first 6 months or between months 6 and 12.

### 2.3 Clinical and anthropometric data

Baseline demographic and clinical variables extracted from medical records included age, sex, body weight, height, body mass index (BMI), presence of type 2 diabetes mellitus (T2DM), previous exposure to glucagon-like peptide-1 receptor agonists (GLP-1 RAs), and concomitant statin therapy.

Body weight was recorded at baseline and at approximately 6 and 12 months, when available as part of routine clinical follow-up. BMI was calculated as body weight in kilograms divided by height in meters squared (kg/m^2^). Absolute weight change was calculated as the difference between baseline and follow-up body weight, whereas percentage weight loss was calculated relative to baseline body weight.

Weight-loss effectiveness was assessed both continuously and according to the proportions of patients achieving ≥5%, ≥10%, ≥15%, ≥20%, ≥25%, and ≥30% reductions from baseline body weight. Among patients with measurements available at both 6 and 12 months, additional weight change during the second 6 months of treatment was also evaluated.

Tirzepatide dose was extracted from the clinical records at 6 and 12 months. For analyses examining treatment intensification during the second half of follow-up, dose escalation was defined as a higher tirzepatide dose at month 12 than at month 6. Patients receiving the same or a lower dose at month 12 were classified as having no dose escalation.

### 2.4 Metabolic and biochemical assessments

Available laboratory measurements obtained during routine clinical care were retrospectively extracted at baseline and at approximately 6 and 12 months following tirzepatide initiation. The parameters evaluated included glycated hemoglobin (HbA1c), low-density lipoprotein cholesterol (LDL-C), high-density lipoprotein cholesterol (HDL-C), triglycerides, aspartate aminotransferase (AST), alanine aminotransferase (ALT), γ-glutamyl transferase (γ-GT), and estimated glomerular filtration rate (eGFR).

Laboratory investigations were performed as part of routine clinical care rather than according to a study-mandated testing schedule. Consequently, laboratory measurements were not available for every patient at every time point, and the number of observations varied according to the parameter and follow-up assessment. No additional laboratory testing was performed specifically for the purposes of the present retrospective study.

### 2.5 Study outcomes

The principal outcomes of the present 12-month analysis were documented tirzepatide treatment persistence at 12 months, confirmed treatment discontinuation and reasons for discontinuation, and change in body weight through 12 months. Treatment persistence was assessed separately from loss to follow-up to avoid classifying patients with unknown treatment status as having discontinued treatment.

Additional effectiveness outcomes included absolute and percentage weight loss at 6 and 12 months; achievement of ≥5%, ≥10%, ≥15%, ≥20%, ≥25%, and ≥30% weight-loss thresholds; additional weight change between months 6 and 12; tirzepatide dose distribution and dose trajectory; and longitudinal changes in metabolic and biochemical parameters.

The association between early treatment response and subsequent 12-month effectiveness was also investigated. Six-month percentage weight loss was evaluated in relation to additional percentage weight loss between months 6 and 12. Achievement of ≥20% cumulative weight loss at month 12 was also evaluated among participants who had not already achieved this threshold at month 6. The association between dose escalation during months 6–12 and subsequent weight loss was examined as an exploratory outcome.

To characterize weight trajectories during the second half of the observation period, patients with body-weight measurements available at both 6 and 12 months were categorized according to their change relative to body weight at month 6. Continued weight loss was defined as a further reduction of >2%, weight stability as a change within ±2%, and weight regain as an increase of >2%.

### 2.6 Data collection and quality control

Data were retrospectively extracted from routine clinical records at the participating centers and compiled into a standardized study database. Prior to analysis, the dataset underwent systematic quality control to assess completeness, plausibility, internal consistency, and duplicate records. Anthropometric measurements, treatment dose, laboratory measurements, treatment status, and documented reasons for discontinuation were cross-checked where appropriate.

Particular attention was paid to distinguishing confirmed treatment discontinuation from loss to follow-up. Discontinuation was recorded only when cessation of tirzepatide was documented in the available clinical information. When treatment status could not be reliably established, the patient was classified as lost to follow-up rather than as having discontinued treatment.

Data availability was assessed separately for each variable and follow-up time point. Missing clinical or laboratory measurements were not replaced by assumed values, and denominators therefore varied across individual outcome analyses according to data availability.

### 2.7 Ethical considerations

The study was conducted in accordance with the principles of the Declaration of Helsinki and was approved by the Ethics Committee of the Hellenic Endocrine Network (Protocol No. N2024/0121316). The approval covered the retrospective collection and analysis of the 12-month clinical data included in the present study. All patient-level data were anonymized before analysis and handled in accordance with applicable data-protection requirements. The Ethics Committee explicitly waived the requirement for informed consent due to the fully anonymized and retrospective nature of the study.

### 2.8 Statistical analysis

Continuous variables are presented as mean ± standard deviation (SD) and categorical variables as frequencies and percentages, unless otherwise specified. Distributional assumptions were assessed before inferential analyses. Paired-samples t tests or Wilcoxon signed-rank tests were used for within-participant comparisons between two time points, and independent-samples t tests or Mann–Whitney U tests for comparisons between two independent groups, as appropriate. Comparisons across three independent groups were performed using one-way analysis of variance (ANOVA) or the Kruskal–Wallis test. Categorical variables were compared using Fisher's exact test, while distributions of reasons for treatment discontinuation across periods were compared using the Fisher–Freeman–Halton exact test.

Treatment persistence was evaluated descriptively at the prespecified 6- and 12-month assessments, with confirmed discontinuations and loss to follow-up considered separately. The primary effectiveness outcome was on-treatment weight change from baseline to 12 months, assessed among patients with available weight measurements obtained while receiving tirzepatide. An additional available-case sensitivity analysis was restricted to participants without previous GLP-1 RA exposure, with descriptive comparison against participants with previous exposure.

The 12-month analysis included 180 participants with paired baseline and 12-month weight measurements obtained while still receiving tirzepatide. Of these, 170 continued treatment beyond the 12-month visit, whereas 10 discontinued treatment following their weight assessment at that visit. No post-discontinuation weight measurements or imputed values were included in the available-case analysis. Analyses of weight change between months 6 and 12 included 179 participants with measurements available at both follow-up assessments.

The association between dose escalation from months 6–12 and additional percentage weight loss was examined using multivariable linear regression adjusted for age, sex, type 2 diabetes mellitus (T2DM), previous GLP-1 receptor agonist (GLP-1 RA) exposure, baseline body mass index (BMI), and 6-month percentage weight loss.

Longitudinal changes in metabolic and biochemical parameters were assessed using linear mixed-effects models (LMMs), with time as a categorical fixed effect and participant as a random intercept. Post-hoc pairwise comparisons were Holm-adjusted for multiple testing. Sensitivity analyses using non-parametric paired tests were performed for selected outcomes with non-normal distributions.

To assess the prognostic value of early treatment response while avoiding mathematical coupling with cumulative 12-month weight loss, additional percentage weight loss between months 6 and 12 was calculated as the difference between 12-month and 6-month percentage weight loss, both expressed relative to baseline body weight. Its association with 6-month percentage weight loss was evaluated using Pearson correlation and multivariable linear regression adjusted for age, sex, T2DM, previous GLP-1 RA exposure, and baseline BMI. To evaluate achievement of ≥20% cumulative weight loss at month 12, a 6-month landmark analysis was performed among participants who had not already achieved this threshold at month 6. Multivariable logistic regression included 6-month percentage weight loss and the same covariates. Discrimination was assessed using receiver operating characteristic (ROC) analysis, with bootstrap 95% confidence intervals for the area under the curve (AUC) and the optimal threshold determined using the Youden index. Model assumptions and collinearity were assessed using standard diagnostic procedures.

Primary analyses were performed on an available-case basis. To assess the potential impact of missing 12-month weight measurements, multiple imputation by chained equations was performed using 40 imputed datasets under a missing-at-random (MAR) assumption. The imputation model included age, sex, baseline weight, baseline BMI, T2DM, previous GLP-1 RA exposure, statin use, and 6-month percentage weight loss. Missing 6-month responses were also imputed. Imputation was performed using Bayesian ridge regression with posterior sampling. Estimates were combined using Rubin's rules. Delta-adjusted sensitivity analyses additionally assumed that participants without 12-month measurements experienced 3, 5, or 10 percentage points less weight loss than predicted under MAR. LOCF and BOCF were retained as supplementary scenario-based analyses. These analyses evaluated hypothetical missing outcomes in the original cohort and were not considered equivalent to the primary on-treatment effectiveness analysis.

Additional sensitivity analyses excluded participants with T2DM and repeated the multivariable linear and logistic regression models and ROC analysis. All tests were two-sided, with P < 0.05 considered statistically significant. Statistical analyses were performed using MedCalc Statistical Software version 23.7 (MedCalc Software Ltd., Ostend, Belgium). Multiple imputation and delta-adjusted sensitivity analyses were performed using Python with the scikit-learn library (IterativeImputer with BayesianRidge estimation and posterior sampling).

## 3 Results

### 3.1 Baseline characteristics

The study population comprised 249 adults treated with tirzepatide in routine clinical practice. The mean age was 49.2 ± 10.8 years, and 163 participants (65.5%) were women. Mean baseline body weight was 113.6 ± 28.5 kg, corresponding to a mean BMI of 39.2 ± 8.2 kg/m^2^. Type 2 diabetes was present in 34 participants (13.7%), while 83 (33.3%) had previously received a GLP-1 receptor agonist and 83 (33.3%) were receiving statin therapy.

Among participants with previous exposure to GLP-1 receptor agonist therapy (n = 83), the most commonly used agents were semaglutide (n = 44) and liraglutide for obesity treatment (n = 32), while previous exposure to other GLP-1 receptor agonists was uncommon (Table 1). Participants were recruited from 13 clinical practices, with individual center contributions ranging from 6 to 56 participants. The distribution of participants across centers is presented in Supplementary Table S1.

**Table 1:** Baseline demographic and clinical characteristics of the study population.

Characteristic	Overall population (N = 249)
Age, years	49.2 ± 10.8
Female sex, n (%)	163 (65.5)
Male sex, n (%)	86 (34.5)
Type 2 diabetes, n (%)	34 (13.7)
Previous GLP-1 receptor agonist use, n (%)	83 (33.3)
Statin therapy, n (%)	83 (33.3)
Body weight, kg	113.6 ± 28.5; median 110.0 (IQR 93.3–126.0)
BMI, kg/m^2^	39.2 ± 8.2; median 38.1 (IQR 33.2–43.3)
HbA1c, %	5.61 ± 0.57
LDL cholesterol, mg/dL	110.1 ± 32.9
HDL cholesterol, mg/dL	50.0 ± 11.9
Triglycerides, mg/dL	117.0 ± 54.4
AST, U/L	22.5 ± 8.8
ALT, U/L	29.1 ± 18.5
γ-GT, U/L	30.8 ± 20.1

Data are presented as mean ± SD or n (%), as appropriate; for baseline body weight and BMI, which showed right-skewed distributions, median and interquartile range (IQR) are additionally reported.

### 3.2 Treatment persistence and discontinuation

At 6 months, continued tirzepatide treatment was documented in 203 of 249 participants (81.5%), while 43 (17.3%) had confirmed treatment discontinuation and 3 (1.2%) were lost to follow-up. Between months 6 and 12, an additional 21 participants discontinued treatment and 12 were lost to follow-up. Consequently, following the 12-month assessment, 170 participants (68.3% of the original cohort) continued tirzepatide treatment, while 64 (25.7%) had confirmed treatment discontinuation, including 10 who discontinued following their weight assessment at the 12-month visit, and 15 (6.0%) were lost to follow-up (Fig. 1).

**Fig. 1:**
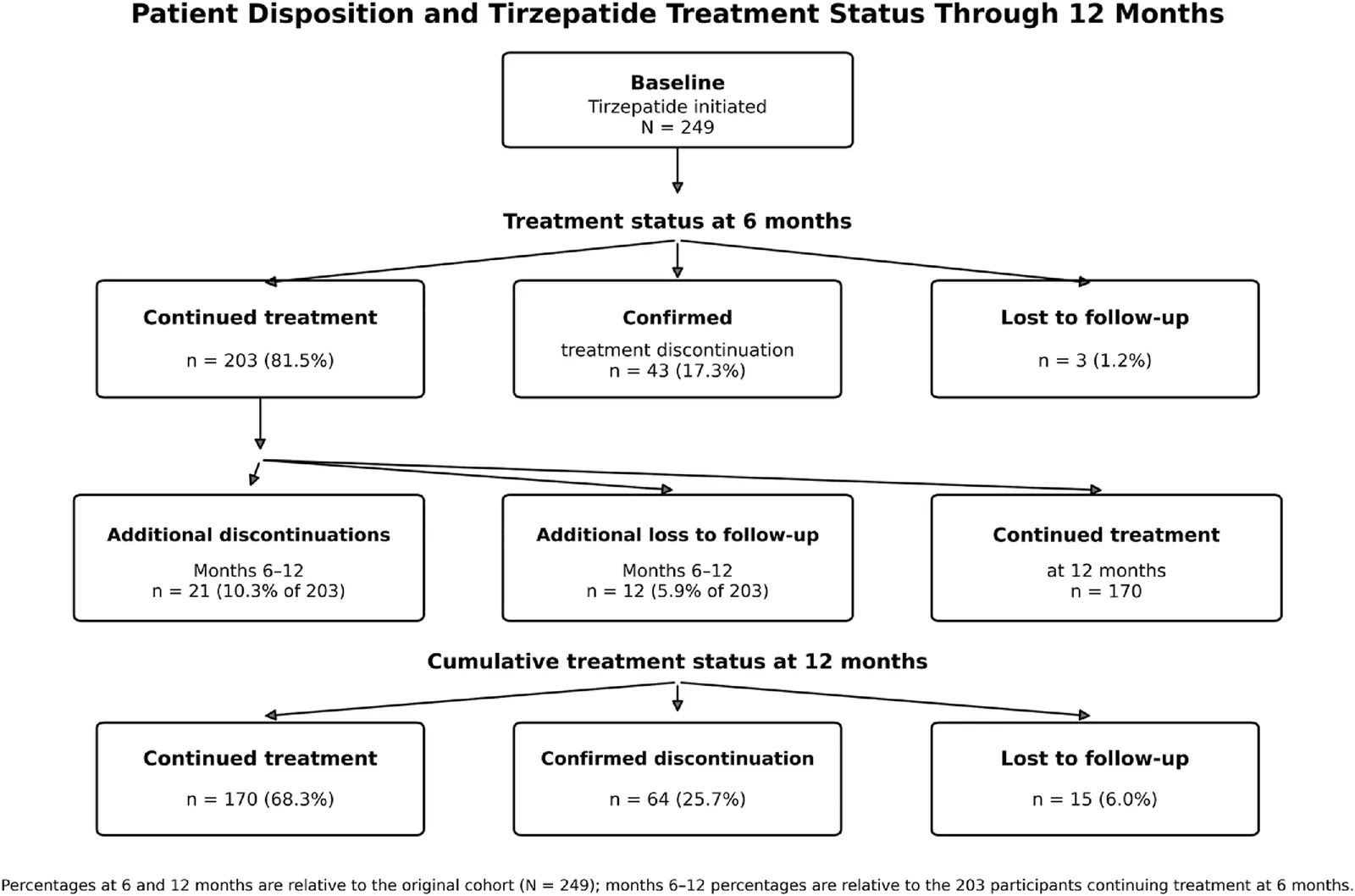
**Patient disposition and tirzepatide treatment status through 12 months**. Treatment status was assessed at the prespecified 6- and 12-month follow-up points. Confirmed treatment discontinuations and participants lost to follow-up are presented separately. Percentages at 6 and 12 months were calculated relative to the original study cohort (N = 249); percentages for additional events occurring between months 6 and 12 were calculated among participants continuing treatment at 6 months (n = 203).

### 3.3 Reasons for treatment discontinuation

Among the 64 participants with confirmed treatment discontinuation during the 12-month observation period, achievement of the treatment goal was the most frequently reported reason (21/64, 32.8%), followed by adverse events (17/64, 26.6%), treatment cost (14/64, 21.9%), and inadequate treatment response (12/64, 18.8%).Achievement of the treatment goal was defined according to the individual patient's satisfaction with the weight loss achieved, as assessed in consultation with the treating physician. No uniform target weight or predefined percentage weight-loss threshold was applied, reflecting individualized decision-making in routine clinical practice. Among the 17 participants who discontinued treatment because of adverse events, the documented events were diarrhea (n = 6), increased calcitonin levels (n = 3), injection-site reactions (n = 2), nausea (n = 2), cholecystitis (n = 1), hypoglycemia (n = 1), panic attacks (n = 1), and abdominal pain (n = 1).In the three patients who discontinued tirzepatide because of elevated calcitonin levels, the decision was based on a predefined precautionary threshold of >12 pg/mL, irrespective of baseline calcitonin concentrations. These discontinuations reflected a clinical safety precaution rather than an established causal relationship between tirzepatide and calcitonin elevation. No serious adverse events were identified in the available clinical records during the 12-month observation period, including no documented cases of pancreatitis or adverse events requiring hospitalization. The relative contribution of adverse events decreased numerically from 30.2% of discontinuations during the first 6 months to 19.0% between months 6 and 12, whereas achievement of the treatment goal increased from 27.9% to 42.9%. However, the overall distribution of reasons for treatment discontinuation did not differ significantly between the two periods (Fisher–Freeman–Halton exact test, P = 0.605, Table 2).

**Table 2:** Reasons for confirmed tirzepatide discontinuation according to treatment period.

Reason for discontinuation	≤6 months (n = 43)	6–12 months (n = 21)	Overall (n = 64)
Adverse events	13 (30.2%)	4 (19.0%)	17 (26.6%)
Achievement of treatment goal	12 (27.9%)	9 (42.9%)	21 (32.8%)
Cost	9 (20.9%)	5 (23.8%)	14 (21.9%)
Inadequate response	9 (20.9%)	3 (14.3%)	12 (18.8%)
**Total**	**43 (100%)**	**21 (100%)**	**64 (100%)**

The overall distribution of reasons for discontinuation did not differ significantly between treatment periods (Fisher–Freeman–Halton exact test, P = 0.605).

### 3.4 Weight-loss effectiveness through 12 months

Among participants with available paired measurements (n = 223), mean body weight decreased from 114.9 ± 29.2 kg at baseline to 98.2 ± 26.9 kg at 6 months, corresponding to a mean absolute weight reduction of 16.7 ± 8.6 kg and a mean percentage weight loss of 14.6 ± 6.5% (P < 0.001).

Among participants with available paired baseline and 12-month body-weight measurements obtained while receiving tirzepatide (n = 180), mean body weight decreased from 117.0 ± 30.3 kg to 93.4 ± 25.7 kg, corresponding to a mean absolute weight reduction of 23.6 ± 12.5 kg and a mean percentage weight loss of 19.9 ± 8.6% (P < 0.001). In a sensitivity analysis restricted to participants without previous GLP-1 RA exposure (n = 166 at baseline), 122 had available 12-month weight measurements and achieved a mean weight loss of 20.3 ± 8.3%. Among participants with previous GLP-1 RA exposure, 58 of 83 had available 12-month measurements, with a mean weight loss of 18.9 ± 9.4%. These descriptive findings should be interpreted cautiously because the groups were not randomized and may differ in baseline characteristics and previous treatment history.

Of the 69 participants without 12-month weight measurements, 54 had discontinued treatment before the assessment and 15 were lost to follow-up. Participants with available 12-month measurements had higher baseline weight (117.0 vs. 104.7 kg; P < 0.001) and BMI (40.0 vs. 37.2 kg/m^2^; P = 0.008), whereas 6-month percentage weight loss did not differ significantly (15.0% vs. 13.3%; P = 0.133; Supplementary Table S2).

In sensitivity analyses addressing missing 12-month weight measurements, multiple imputation under the MAR assumption yielded an estimated mean weight loss of 19.5% (95% CI 18.3–20.7%) in the baseline cohort (N = 249). Under delta-adjusted scenarios assuming 3, 5, and 10 percentage points less weight loss among participants without 12-month measurements, the corresponding estimates were 18.6%, 18.1%, and 16.7%, respectively. The previously calculated LOCF and BOCF estimates were 16.7% and 14.4%, respectively. These analyses depend on unverifiable assumptions regarding missing outcomes, particularly following treatment discontinuation, and should not be interpreted as unbiased estimates of treatment effectiveness in the full cohort.

At 12 months, 95.0%, 88.9%, 73.3%, and 50.0% of participants with available weight measurements achieved ≥5%, ≥10%, ≥15%, and ≥20% weight loss, respectively, whereas 27.2% and 8.9% achieved ≥25% and ≥30% weight loss, respectively.

Among the 179 participants with weight measurements available at baseline, 6 months, and 12 months, body weight decreased further from 99.7 kg at 6 months to 93.5 kg at 12 months, corresponding to an additional mean reduction of 6.2 ± 8.2 kg (P < 0.001, Table 3).

**Table 3:** Achievement of clinically relevant weight-loss thresholds at 6 and 12 months.

Weight-loss threshold	6 months (n = 223)	12 months (n = 180)
≥5%	206 (92.4%)	171 (95.0%)
≥10%	172 (77.1%)	160 (88.9%)
≥15%	108 (48.4%)	132 (73.3%)
≥20%	45 (20.2%)	90 (50.0%)
≥25%	14 (6.3%)	49 (27.2%)
≥30%	2 (0.9%)	16 (8.9%)

Percentages were calculated among participants with available body-weight measurements at each assessment point (n = 223 at 6 months and n = 180 at 12 months).

In a sensitivity analysis of participants with available baseline and 12-month weight measurements (n = 180), we examined potential center effects using multivariable linear regression adjusted for age, sex, T2DM, previous GLP-1 RA exposure, and baseline BMI. The addition of clinical practice as a categorical variable significantly improved model fit (F[12,162] = 2.36, P = 0.008), with adjusted R^2^ increasing from 0.081 to 0.160. These findings suggest between-center heterogeneity in 12-month weight-loss outcomes, although interpretation is limited by the small number of participants with complete outcome data at some centers.

### 3.5 Tirzepatide dose trajectory

Tirzepatide dose escalation continued throughout the 12-month treatment period. Among participants with available dose information at 6 months (n = 204), the mean dose was 7.34 mg and the median dose was 7.5 mg. Most participants remained on relatively moderate doses, with 70.1% receiving ≤7.5 mg and only 3.9% receiving ≥12.5 mg.

At 12 months, among participants with available dose information (n = 165), the mean tirzepatide dose had increased to 9.71 mg, with a median dose of 10 mg. Overall, 38.2% remained on doses ≤7.5 mg, whereas 31.5% were receiving 12.5 or 15 mg.

Among the 160 participants with dose information available at both 6 and 12 months, 95 (59.4%) underwent further dose escalation, 53 (33.1%) remained on the same dose, and 12 (7.5%) received a lower dose at 12 months. The mean within-participant increase in tirzepatide dose between months 6 and 12 was 2.42 ± 3.35 mg (*P* < 0.001).

### 3.6 Dose escalation and weight-loss response

Among 159 of these participants with body-weight measurements also available at both 6 and 12 months, 94 underwent further tirzepatide dose escalation, whereas 65 remained on the same or a lower dose. Participants undergoing dose escalation experienced greater additional weight loss between months 6 and 12 than those without further escalation (8.1 ± 8.5 vs 4.6 ± 6.5 kg; P = 0.004), corresponding to an additional loss of 6.4 ± 6.0% versus 4.1 ± 5.6% of baseline body weight, respectively (P = 0.014) (Table 4).

**Table 4:** Tirzepatide dose trajectory and weight-loss outcomes according to dose escalation between 6 and 12 months.

A. Tirzepatide dose distribution		
Tirzepatide dose	6 months (n = 204)	12 months (n = 165)
2.5 mg	8 (3.9%)	7 (4.2%)
5 mg	69 (33.8%)	30 (18.2%)
7.5 mg	66 (32.4%)	26 (15.8%)
10 mg	53 (26.0%)	50 (30.3%)
12.5 mg	5 (2.5%)	16 (9.7%)
15 mg	3 (1.5%)	36 (21.8%)
Mean dose, mg	7.34	9.71
Median dose, mg	7.5	10.0

B. Weight-loss outcomes according to dose escalation			
Outcome	Dose escalation (n = 94)	No escalation (n = 65)	P-value
Additional weight loss, 6–12 months, kg	8.1 ± 8.5	4.6 ± 6.5	0.004
Additional weight loss, 6–12 months, % of baseline weight	6.4 ± 6.0	4.1 ± 5.6	0.014
Total 12-month weight loss, %	21.0 ± 7.9	20.0 ± 8.6	0.467
≥20% weight loss at 12 months	51 (54.3%)	33 (50.8%)	0.747
≥25% weight loss at 12 months	27 (28.7%)	19 (29.2%)	1.000

Data are presented as mean ± SD or n (%), as appropriate. Dose escalation was defined as a higher tirzepatide dose at 12 months than at 6 months; the no-escalation group included participants receiving the same or a lower dose at 12 months. Dose-distribution percentages were calculated among participants with available dose information at each assessment point.

Despite this difference in second-half treatment response, total percentage weight loss at 12 months was similar between participants with and without dose escalation (21.0 ± 7.9% vs 20.0 ± 8.6%; P = 0.467). Likewise, the proportions achieving ≥20% (54.3% vs 50.8%; P = 0.747) or ≥25% weight loss (28.7% vs 29.2%; P = 1.000) did not differ significantly between groups.

In an exploratory multivariable analysis adjusting for age, sex, type 2 diabetes, previous GLP-1 receptor agonist exposure, baseline BMI, and 6-month weight response, dose escalation was not independently associated with additional percentage weight loss between months 6 and 12 (β = 1.61 percentage points, 95% CI −0.44 to 3.66; P = 0.123).

Furthermore, total 12-month percentage weight loss did not differ significantly according to the tirzepatide dose being administered at month 12 (19.8 ± 7.9% for ≤7.5 mg, 21.8 ± 9.2% for 10 mg, and 20.6 ± 7.7% for ≥12.5 mg; P = 0.429).

### 3.7 Metabolic and biochemical outcomes

Significant longitudinal changes were observed in HbA1c, LDL cholesterol, triglycerides, and hepatic biochemical markers over the 12-month treatment period (all P for time <0.01). Mean HbA1c decreased from 5.61 ± 0.57% at baseline to 5.31 ± 0.44% at 6 months and 5.21 ± 0.40% at 12 months (P for time <0.001). Notably, HbA1c continued to decrease significantly between months 6 and 12 (adjusted P < 0.001).

LDL cholesterol decreased from 110.1 ± 32.9 mg/dL at baseline to 99.9 ± 31.0 mg/dL at 6 months and remained reduced at 98.2 ± 29.7 mg/dL at 12 months (P for time <0.001), with no further significant change between months 6 and 12 (adjusted P = 0.529). Triglycerides decreased progressively from 117.0 ± 54.4 to 103.3 ± 41.4 and 98.8 ± 41.2 mg/dL, respectively (P for time <0.001), with a further significant reduction between months 6 and 12 (adjusted P = 0.026). No significant overall longitudinal change was observed in HDL cholesterol (P for time = 0.084).

AST, ALT, and γ-GT also demonstrated significant overall reductions during follow-up (P for time = 0.006, <0.001, and <0.001, respectively). However, no further significant changes in these hepatic markers were observed between months 6 and 12, suggesting that the improvements achieved during the first 6 months were subsequently maintained. Renal function remained stable throughout follow-up, with no significant longitudinal change in eGFR (P for time = 0.060).

Sensitivity analyses using log-transformed values for triglycerides and γ-GT yielded consistent results. The overall time effect remained significant for both parameters (P < 0.001), while the reduction in triglycerides between 6 and 12 months remained significant after adjustment for multiple comparisons (P = 0.020); no significant change in γ-GT was observed between 6 and 12 months (P = 0.284) (Table 5).

**Table 5:** Longitudinal changes in metabolic and biochemical parameters during 12 months of tirzepatide treatment.

Parameter	Baseline	6 months	12 months	Baseline-to-12-month change (95% CI)	P for overall time effect	6 vs 12 mo, adjusted P
HbA1c, %	5.61 ± 0.57(235)	5.31 ± 0.44(199)	5.21 ± 0.40(163)	**−0.42 (−0.49 to −0.35)**	<0.001	<0.001
LDL-C, mg/dL	110.1 ± 32.9(242)	99.9 ± 31.0 (219)	98.2 ± 29.7 (169)	**−12.86 (−17.20 to −8.52)**	<0.001	0.529
HDL-C, mg/dL	50.0 ± 11.9 (243)	49.3 ± 14.0 (219)	50.6 ± 15.2 (169)	**+2.58 (+0.48 to +4.68)**	0.084	0.142
Triglycerides, mg/dL	117.0 ± 54.4 (243)	103.3 ± 41.4 (220)	98.8 ± 41.2 (167)	**−22.14 (−29.86 to −14.43)**	<0.001	0.026
AST, U/L	22.5 ± 8.8 (245)	21.2 ± 8.3 (225)	20.9 ± 7.2 (172)	**−1.63 (−2.90 to −0.35)**	0.006	0.638
ALT, U/L	29.1 ± 18.5 (243)	24.9 ± 13.2 (225)	24.7 ± 12.3 (171)	**−5.25 (−7.76 to −2.74)**	<0.001	0.909
γ-GT, U/L	30.8 ± 20.1 (223)	23.9 ± 14.2 (210)	25.1 ± 12.6 (165)	**−7.03 (−9.86 to −4.20)**	<0.001	0.226
eGFR, mL/min/1.73m^2^	104.4 ± 30.9 (242)	103.0 ± 28.4 (223)	104.3 ± 28.3 (163)	**−3.38 (−6.67 to −0.08)**	0.060	0.425

Data at each time point are presented as mean ± SD, with the number of available observations (n) indicated in parentheses. Sample sizes vary across laboratory parameters and assessment time points because measurements were obtained during routine clinical care and were not available for all participants at every visit. Baseline-to-12-month changes are mean within-participant changes with 95% confidence intervals calculated among participants with measurements available at both time points; negative values indicate a decrease from baseline. Overall changes across baseline, 6 months, and 12 months were assessed using linear mixed-effects models (LMMs), incorporating all available measurements and accounting for within-participant correlation across repeated observations. The P value for time represents the overall longitudinal effect across the three assessment points. Pairwise comparisons between time points were performed using paired tests, with P values adjusted for multiple comparisons using the Holm method; the final column reports the adjusted P value for the 6- versus 12-month comparison. Statistical significance was defined as a two-sided P < 0.05. HbA1c, glycated hemoglobin; LDL-C, low-density lipoprotein cholesterol; HDL-C, high-density lipoprotein cholesterol; AST, aspartate aminotransferase; ALT, alanine aminotransferase; γ-GT, gamma-glutamyl transferase; eGFR, estimated glomerular filtration rate; CI, confidence interval; SD, standard deviation; LMM, linear mixed-effects model.

### 3.8 Early weight loss and subsequent weight-loss outcomes

Among 179 participants with available weight measurements at baseline, 6 months, and 12 months, percentage weight loss achieved during the first 6 months was not significantly correlated with additional percentage weight loss between months 6 and 12 (r = −0.090, P = 0.229).

In multivariable linear regression adjusted for age, sex, T2DM, previous GLP-1 RA exposure, and baseline BMI, 6-month percentage weight loss was not independently associated with additional weight loss during months 6–12 (β = −0.110, 95% CI −0.255 to 0.035; P = 0.135). Higher baseline BMI was independently associated with greater additional weight loss (β = 0.215, 95% CI 0.106–0.325; P < 0.001). The model explained a limited proportion of the variability in additional weight loss (adjusted R^2^ = 0.067) (Table 6, Panel A).

**Table 6:** Multivariable predictors of additional weight loss during months 6–12 and achievement of ≥20% cumulative weight loss at month 12.

Panel A. Multivariable linear regression for additional percentage weight loss during months 6–12 (n = 179)		
Predictor	β (95% CI)	P-value
6-month percentage weight loss	−0.110 (−0.255 to 0.035)	0.135
Age (years)	−0.011 (−0.100 to 0.079)	0.817
Female sex	−1.547 (−3.460 to 0.365)	0.112
T2DM	−0.925 (−3.862 to 2.012)	0.535
Previous GLP-1 RA exposure	0.493 (−1.653 to 2.639)	0.651
Baseline BMI (kg/m^2^)	0.215 (0.106–0.325)	<0.001
Adjusted R^2^ = 0.067		

Panel B. Multivariable logistic regression for achievement of ≥20% cumulative weight loss at month 12 among participants below this threshold at month 6 (n = 139)		
Predictor	Adjusted OR (95% CI)	P-value
6-month percentage weight loss	1.34 (1.19–1.51)	<0.001
Age (years)	1.00 (0.96–1.05)	0.818
Female sex	0.49 (0.21–1.19)	0.117
T2DM	0.20 (0.05–0.83)	0.026
Previous GLP-1 RA exposure	2.13 (0.76–5.96)	0.148
Baseline BMI (kg/m^2^)	1.08 (1.03–1.13)	0.003

Data are presented as regression coefficients (β) or adjusted odds ratios (OR), with 95% confidence intervals (CI). Panel A presents multivariable linear regression for additional percentage weight loss between months 6 and 12, calculated as the difference between cumulative percentage weight loss at months 12 and 6 (n = 179). Panel B presents multivariable logistic regression for achievement of ≥20% cumulative weight loss at month 12 among participants who had not achieved this threshold at month 6 (n = 139). Both models were adjusted for age, sex, T2DM, previous GLP-1 RA exposure, and baseline BMI. Male sex, absence of T2DM, and no previous GLP-1 RA exposure were the reference categories. Regression coefficients for continuous predictors represent the change associated with a one-unit increase in the predictor; odds ratios for continuous predictors are expressed per one-unit increase.

#### 3.8.1 Six-month landmark analysis for achievement of ≥20% weight loss at 12 months

Of the 179 participants with available weight measurements at baseline, 6 months, and 12 months, 40 had already achieved ≥20% weight loss at month 6 and were excluded from the landmark analysis. Among the remaining 139 participants, 52 (37.4%) achieved ≥20% cumulative weight loss at month 12. Six-month percentage weight loss demonstrated moderate discrimination for achieving this threshold (AUC 0.760, 95% bootstrap CI 0.675–0.837). The optimal threshold according to the Youden index was ≥12.5%, corresponding to a sensitivity of 82.7% and specificity of 60.9% (Fig. 2).

**Fig. 2:**
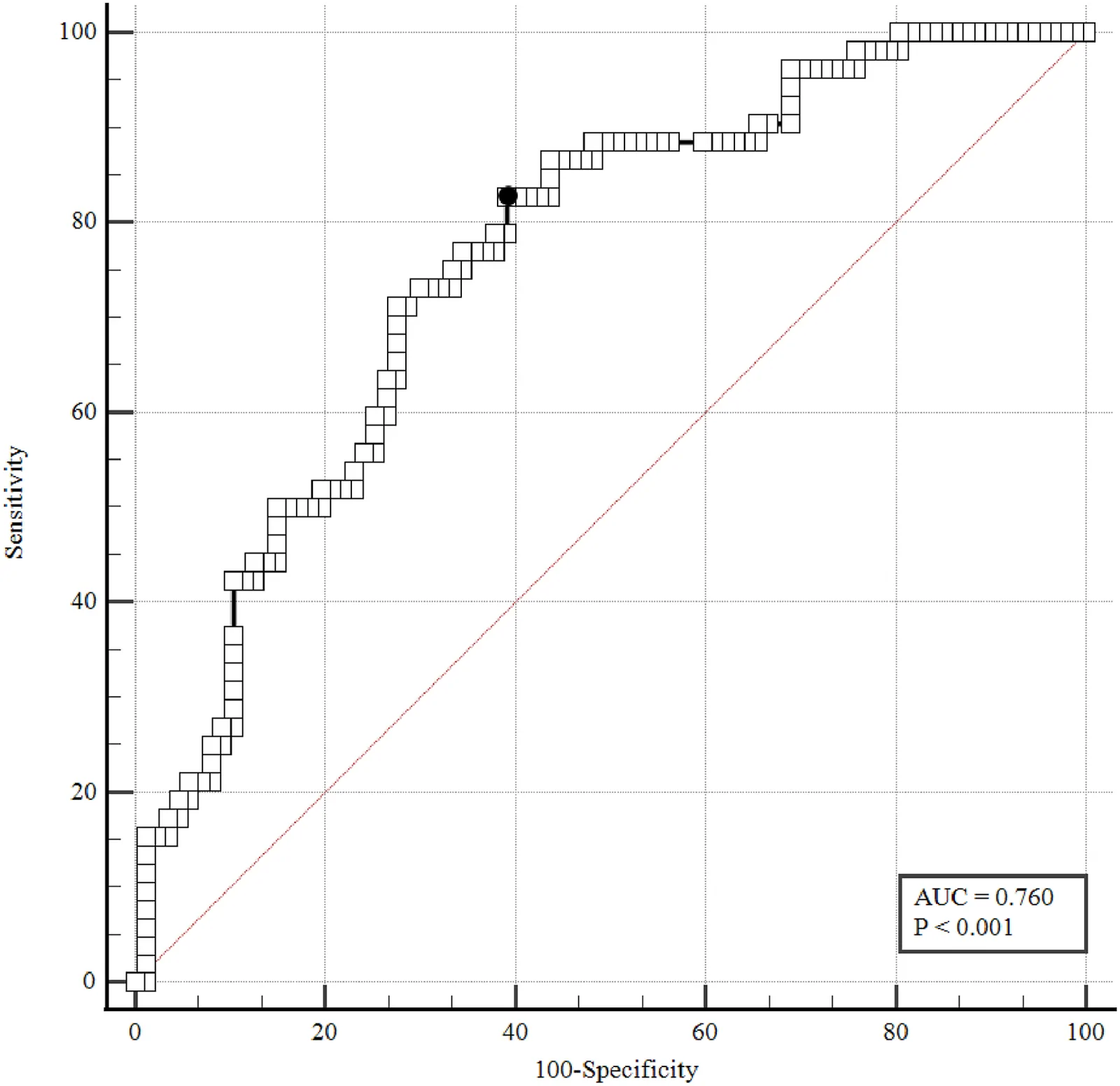
**Receiver operating characteristic curve for 6-month percentage weight loss as a predictor of achieving ≥20% cumulative weight loss at month 12 among participants who had not achieved this threshold at month 6**. Among 139 participants with <20% weight loss at month 6, 6-month percentage weight loss demonstrated moderate discrimination for achieving ≥20% cumulative weight loss at month 12 (AUC 0.760, 95% bootstrap CI 0.675–0.837). The optimal cutoff according to the Youden index was ≥12.5%, corresponding to a sensitivity of 82.7% and specificity of 60.9%.

In multivariable logistic regression adjusted for age, sex, T2DM, previous GLP-1 RA exposure, and baseline BMI, each additional percentage point of weight loss at month 6 was associated with higher odds of achieving ≥20% cumulative weight loss at month 12 (adjusted OR 1.34, 95% CI 1.19–1.51; P < 0.001). Higher baseline BMI was also associated with achieving this threshold (adjusted OR 1.08 per kg/m^2^, 95% CI 1.03–1.13; P = 0.003), whereas T2DM was associated with lower odds (adjusted OR 0.20, 95% CI 0.05–0.83; P = 0.026) (Table 6, Panel B).

### 3.9 Sensitivity analysis excluding participants with type 2 diabetes

Given the independent association between T2DM and a lower likelihood of achieving ≥20% weight loss, sensitivity analyses were performed after excluding participants with T2DM. At 12 months, mean percentage weight loss was 20.1 ± 8.9% among participants without T2DM (n = 156), compared with 18.1 ± 6.8% among those with T2DM (n = 24; P = 0.207). The proportion achieving ≥20% weight loss was higher among participants without T2DM than among those with T2DM (53.2% vs 29.2%; P = 0.047).

In the sensitivity analysis excluding participants with T2DM, 6-month percentage weight loss was not independently associated with additional percentage weight loss between months 6 and 12 (β = −0.028, 95% CI −0.185 to 0.129; P = 0.722). In the corresponding landmark analysis, 118 participants without T2DM had not achieved ≥20% weight loss at month 6, of whom 48 (40.7%) achieved this threshold at month 12. Six-month percentage weight loss demonstrated moderate discrimination (AUC 0.785), with an optimal cutoff of ≥12.5% (sensitivity 85.4%, specificity 62.9%). The association remained significant after multivariable adjustment for age, sex, previous GLP-1 RA exposure, and baseline BMI (adjusted OR 1.39 per additional percentage point of 6-month weight loss, 95% CI 1.22–1.59; P < 0.001).

### 3.10 Weight trajectory between 6 and 12 months

Among the 179 participants with body-weight measurements available at both 6 and 12 months, 126 (70.4%) experienced continued weight loss during the second 6 months of treatment, defined as a further reduction of >2% of 6-month body weight. Thirty-five participants (19.6%) exhibited weight stability, defined as a change within ±2%, whereas 18 (10.1%) experienced weight regain of >2%.

Mean body-weight change between months 6 and 12 was −9.6 ± 7.1 kg among participants with continued weight loss, −0.1 ± 1.0 kg among those with weight stability, and +5.9 ± 4.4 kg among those experiencing weight regain. Corresponding total percentage weight loss at 12 months was 22.4 ± 7.5%, 16.0 ± 7.0%, and 10.4 ± 9.9%, respectively.

Six-month percentage weight loss did not differ significantly among the three subsequent weight-trajectory groups (P = 0.505), indicating that the magnitude of early weight loss alone did not distinguish participants who would continue to lose weight from those who subsequently plateaued or regained weight. Baseline BMI differed significantly across trajectory groups (P = 0.003), with the highest baseline BMI observed among participants who continued to lose weight between months 6 and 12. No significant differences were observed according to age, sex, type 2 diabetes, or previous GLP-1 receptor agonist exposure.

## 4 Discussion

The present study provides 12-month real-world evidence on tirzepatide treatment under conditions that differ substantially from those of randomized clinical trials, including individualized dose escalation and entirely self-funded treatment. Three findings appear particularly relevant. First, substantial weight loss was maintained through 12 months, with a mean reduction of 19.9% from baseline, while more than two-thirds of the original cohort had documented treatment persistence. Second, weight loss frequently continued beyond the initial 6 months: among patients with measurements available at both time points, 70.4% experienced a further >2% reduction in body weight between months 6 and 12, whereas only 10.1% experienced >2% weight regain.

Third, 6-month weight loss was associated with the likelihood of reaching ≥20% cumulative weight loss at month 12 among participants who had not already achieved this threshold, but it was not independently associated with the magnitude of additional weight loss between months 6 and 12. Subsequent dose escalation was also not independently associated with additional weight loss after adjustment for relevant clinical factors. Collectively, these findings suggest that substantial and continuing weight loss can be achieved in routine clinical practice despite heterogeneous, non-protocol-driven dosing, while underscoring the need for caution when interpreting early response as a predictor of subsequent weight loss.

The magnitude of 12-month weight loss observed in our cohort compares favorably with emerging real-world evidence. In an academic obesity clinic, Samuels et al. reported a median weight loss of 14.4% among patients who persisted with semaglutide or tirzepatide for at least 12 months, emphasizing the close relationship between persistence and treatment effectiveness [8]. More recently, a multicenter US study reported mean 12-month total body weight loss of 16.6% with tirzepatide overall and 18.5% among patients without diabetes [5]. Other contemporary real-world cohorts have reported weight reductions approaching those observed in randomized trials despite considerable heterogeneity in patient populations, treatment programmes, retention, and dosing patterns [4], [5], [6]. These observations place the 19.9% reduction observed in our cohort toward the upper range of currently reported real-world estimates. Importantly, this degree of weight loss was achieved without protocol-mandated escalation to higher tirzepatide doses, reinforcing previous evidence that substantial effectiveness can be achieved under individualized dosing patterns. Our preceding multicenter real-world studies similarly demonstrated substantial short-term weight loss and metabolic improvement with tirzepatide in routine Greek clinical practice, and the present findings extend these observations through 12 months [9], [10], [11].

Treatment persistence represents an equally important component of real-world effectiveness. In the present cohort, 68.3% of patients had documented continuation of tirzepatide at 12 months, whereas 25.7% had confirmed discontinuation and only 6.0% were lost to follow-up. This distinction is important because absence of follow-up should not automatically be interpreted as pharmacological discontinuation. Persistence with incretin-based anti-obesity therapy has been substantially lower in several real-world datasets, and even within specialist obesity services treatment continuation is frequently incomplete. Samuels et al. reported a median treatment persistence of approximately 11 months in an academic obesity clinic, while large US real-world datasets demonstrated considerable attrition together with slower dose escalation than typically observed in clinical trials [4], [8]. The relatively high documented persistence observed in our cohort is particularly notable because tirzepatide was entirely self-funded throughout the observation period, requiring patients to sustain the full financial burden of therapy. Although patient-perceived value was not directly assessed and therefore cannot be inferred from these data, the finding that 68.3% of the original cohort remained on treatment at 12 months indicates that substantial treatment persistence over this period is possible in routine clinical practice, even in the absence of reimbursement. Nevertheless, treatment cost accounted for 21.9% of confirmed discontinuations, indicating that affordability remained an important barrier to sustained treatment for a meaningful proportion of patients. Adverse events accounted for 26.6% of confirmed discontinuations, corresponding to 6.8% of the overall cohort. Notably, this proportion was within the range of adverse event–related treatment discontinuation reported in SURMOUNT-1 (4.3%–7.1% across tirzepatide dose groups), suggesting that treatment discontinuation due to tolerability in routine clinical practice was broadly consistent with that observed under randomized trial conditions [3].

A particularly informative finding was that 6 months did not represent the end of the weight-loss phase for most patients. Among those with body-weight measurements at both 6 and 12 months, 70.4% experienced a further reduction of >2%, whereas 19.6% remained within ±2% of their 6-month weight and only 10.1% experienced >2% weight regain. This observation is consistent with the prolonged weight-loss trajectory observed across the SURMOUNT programme but extends it to routine clinical practice, where dose escalation was individualized rather than protocol driven. In a post hoc analysis specifically examining time to weight plateau in SURMOUNT-1 and SURMOUNT-4, Horn et al. demonstrated substantial heterogeneity in the duration of active weight loss, with higher baseline BMI associated with a later weight plateau and median time to plateau extending to approximately 36 weeks in the higher BMI categories compared with 24–26 weeks in participants with overweight or class I obesity [15]. This observation is particularly relevant to our findings, as patients who continued to lose weight between months 6 and 12 had a significantly higher baseline BMI than those who subsequently remained weight-stable or regained weight. Although the study designs and outcome definitions differ, the consistency of these findings suggests that greater baseline adiposity may be associated with a more prolonged phase of active weight loss. Collectively, these data argue against assuming that an apparent intermediate response represents a definitive weight plateau and support continued assessment of weight trajectory beyond the first 6 months of treatment.

The prognostic interpretation of early weight loss requires careful consideration because weight loss achieved by month 6 is mathematically incorporated into cumulative weight loss at month 12. Consequently, associations between these outcomes may partly reflect mathematical coupling rather than independent prediction of subsequent treatment response. In our revised analysis, 6-month percentage weight loss was not significantly associated with additional weight loss during months 6–12, either before or after multivariable adjustment. This finding indicates that greater early weight loss did not necessarily translate into greater subsequent weight loss during the second 6 months. Nevertheless, among participants who had not already achieved ≥20% weight loss at month 6, early response retained moderate discriminatory ability for attaining this cumulative threshold by month 12 (AUC 0.760). This finding suggests that the magnitude of weight loss at 6 months may help estimate the probability of reaching a clinically meaningful cumulative target, although it should not be interpreted as evidence that early response predicts the magnitude of further weight reduction. These results should be considered alongside the post hoc analysis of SURMOUNT-1 by Ard et al. [13] and the pooled analysis of SURMOUNT-1 and SURMOUNT-2 by Kokkinos et al. [12], which demonstrated that early weight-loss response was associated with longer-term outcomes, while many slower initial responders nevertheless achieved clinically meaningful weight loss with continued treatment. The identified 12.5% threshold should be considered exploratory and hypothesis-generating, as it was derived and evaluated within the same cohort without independent validation. External validation is required before this threshold can be considered for clinical decision-making. Real-world studies have also examined the association between early weight-loss response and subsequent outcomes during tirzepatide treatment [8], [11].

T2DM was associated with a lower likelihood of achieving ≥20% cumulative weight loss at month 12 in the 6-month landmark analysis (adjusted OR 0.20, 95% CI 0.05–0.83; P = 0.026). However, T2DM was not independently associated with the magnitude of additional percentage weight loss between months 6 and 12 when analyzed as a continuous outcome (P = 0.535). Consistent with this finding, mean 12-month weight loss was numerically lower among participants with T2DM than among those without T2DM (18.1% vs 20.1%), while the proportion achieving ≥20% weight loss was substantially lower (29.2% vs 53.2%). This pattern is consistent with findings across the SURMOUNT clinical trial program, in which the magnitude of weight loss observed with tirzepatide was lower among participants with T2DM in SURMOUNT-2 than among those without diabetes in SURMOUNT-1 [3], [16]. However, comparisons across separate trials should be interpreted cautiously, and given the relatively small number of participants with T2DM in our cohort, this finding should be considered exploratory.

The relationship between dose escalation and weight loss warrants particularly cautious interpretation. Although higher tirzepatide doses produce greater average weight reduction in randomized dose-assigned trials, dosing in routine clinical practice is inherently influenced by treatment response, tolerability, patient preference, and, in the present cohort, treatment cost. In our analysis, further dose escalation between months 6 and 12 was not independently associated with additional percentage weight loss after adjustment for baseline BMI, 6-month response, and other clinical characteristics. Importantly, however, treatment dose was neither randomized nor assigned according to a standardized protocol. Consequently, the present observational analysis cannot establish a causal effect—or absence of such—of dose escalation on subsequent weight loss. The observed association is particularly susceptible to confounding by indication, whereby patients with an insufficient response may preferentially undergo dose escalation, whereas those already achieving satisfactory weight loss may remain at lower doses. Accordingly, our findings should not be interpreted as evidence that higher tirzepatide doses provide no additional benefit or that different doses are therapeutically equivalent. Nevertheless, the substantial weight loss observed at 12 months under individualized dosing suggests that clinically meaningful outcomes can be achieved in routine practice without uniform escalation to the maximum tolerated dose, although the comparative effectiveness of different dosing strategies cannot be established from these observational data. The present findings do not establish the comparative effectiveness or economic advantages of lower-dose treatment strategies, which would require prospective evaluation. Rather, these observational findings illustrate the difficulty of estimating causal dose–response relationships from treatment patterns arising in routine clinical practice. Contemporary real-world studies similarly document substantially slower and more heterogeneous tirzepatide titration than the standardized schedules employed in randomized trials [4]. Randomized or appropriately designed prospective comparative studies would be required to determine whether alternative individualized dose-escalation strategies yield comparable weight-loss outcomes over 12 months and beyond.

Away from weight reduction, tirzepatide treatment was accompanied by favorable changes in several cardiometabolic parameters through 12 months. Improvements in glycemic control, lipid parameters, and liver enzymes are consistent with the broad cardiometabolic benefits reported in the SURMOUNT clinical programme [3] and with emerging real-world evidence [17]. These findings also extend our previous observations in Greek patients, in whom tirzepatide treatment was associated with improvements in glucose homeostasis, lipid profile, and indices of hepatic steatosis [10], [11]. Taken together, the present 12-month data suggest that the substantial weight reduction observed under routine-care conditions is accompanied by sustained improvements across multiple metabolic domains.

### 4.1 Limitations

The study also has limitations. Its retrospective observational design and lack of a comparator group preclude causal inference and introduce the possibility of residual confounding and selection bias. Accordingly, the observed weight and metabolic changes cannot be attributed exclusively to tirzepatide, as concurrent lifestyle interventions, other treatments, and selective retention of participants with available follow-up measurements may have contributed to the findings. Comparisons with randomized trials and other real-world cohorts should also be interpreted cautiously because of differences in patient populations, treatment exposure, follow-up, and outcome definitions, particularly the use of available-case on-treatment estimates in the present study. Follow-up and laboratory testing were performed according to routine clinical practice rather than a standardized study protocol, resulting in variable data availability across outcomes and time points.

Primary weight outcomes were based on available-case analyses. Participants with available 12-month weight measurements had significantly higher baseline weight and BMI than those without measurements, indicating potential selection bias. Although 6-month percentage weight loss did not differ significantly between groups, this comparison was limited by incomplete 6-month data among participants without 12-month measurements. LOCF and BOCF analyses provided alternative estimates under different assumptions regarding missing outcomes, but these simple scenario-based approaches do not account for the underlying missing-data mechanism and may introduce bias.

Although multiple imputation and delta-adjusted sensitivity analyses were performed, the MAR assumption cannot be verified, and informative missingness remains possible, particularly among participants who discontinued treatment. Moreover, these analyses represent hypothetical outcomes in the baseline cohort rather than observed on-treatment weight loss. Therefore, these sensitivity analyses cannot eliminate the possibility of attrition-related bias, and the 19.9% available-case estimate should not be interpreted as representative of the entire baseline cohort. Dose escalation was not randomized and was inherently influenced by treatment response, tolerability, patient preference, and cost, creating substantial potential for confounding by indication. Consequently, associations between dose trajectories and subsequent weight loss should be considered exploratory and cannot support causal inference regarding the comparative effectiveness of different tirzepatide doses or escalation strategies. Reasons for discontinuation were derived from clinical documentation and may not capture all factors contributing to treatment cessation. Subsequent treatment strategies after documented discontinuation, including possible treatment reinitiation, lower-dose maintenance, or transition to alternative interventions, were not systematically captured, and no follow-up was available beyond the 12-month observation period. Therefore, long-term weight maintenance among patients who discontinued tirzepatide after achieving their individualized treatment goals could not be assessed. Adverse events were retrospectively ascertained from available clinical records rather than through systematic prospective monitoring. Consequently, underreporting, particularly of mild or transient events, cannot be excluded.

In addition, one-third of participants had previous exposure to GLP-1 receptor agonists. Although the specific previously used agent was recorded, the duration of prior treatment and the interval between its discontinuation and tirzepatide initiation were not systematically available. Consequently, residual confounding related to previous incretin therapy cannot be excluded. An additional sensitivity analysis restricted to GLP-1 RA–naive participants yielded a similar magnitude of 12-month weight loss; however, this analysis was descriptive, restricted to participants with available measurements, and cannot establish comparative effectiveness between treatment-naive patients and those switching from previous GLP-1 RA therapy. Finally, the landmark prediction analysis was restricted to participants with complete 6- and 12-month weight measurements who had not already achieved ≥20% weight loss at month 6. This may limit generalizability and introduce potential selection bias. Furthermore, the optimal 6-month threshold was derived and evaluated within the same cohort, without independent validation. Its discriminatory performance and clinical utility therefore require confirmation in external populations.

From a clinical perspective, 6-month weight loss may help estimate the likelihood of achieving ≥20% cumulative weight loss at 12 months among patients who have not already reached this threshold, but it does not independently predict the magnitude of additional weight loss during months 6–12. Early response should therefore not be used in isolation to guide treatment continuation or discontinuation. Most patients with serial measurements continued to lose weight between 6 and 12 months, supporting continued treatment and longitudinal reassessment when therapy remains effective and well tolerated. Similarly, the absence of an independent association between further dose escalation and subsequent weight loss should not be used to discourage appropriate titration; rather, it highlights the individualized nature of real-world dosing and the limitations of inferring dose–response relationships from observational data.

Furthermore, sensitivity analysis identified significant between-center heterogeneity in 12-month weight-loss outcomes, even after adjustment for available clinical characteristics. This variation may reflect differences in patient populations, prescribing practices, treatment adherence, or follow-up procedures across participating centers. Given the relatively small sample sizes at some sites, these findings should be interpreted cautiously. Residual center-level confounding cannot be excluded and may limit the generalizability of the findings.

## 5 Conclusion

In conclusion, this multicenter real-world study provides evidence of substantial weight loss and documented treatment persistence with tirzepatide over 12 months under individualized dosing and fully self-funded treatment conditions. These findings highlight the importance of longitudinal assessment of treatment response in routine clinical practice. However, the 12-month observation period does not allow conclusions regarding long-term weight maintenance, particularly after treatment discontinuation.

• Substantial weight loss can be sustained through 12 months with tirzepatide in routine clinical practice, even with individualized, non-protocol-driven dose escalation.
• Weight loss commonly continues beyond 6 months, supporting continued longitudinal assessment rather than assuming that an intermediate response represents a definitive plateau.
• Six-month weight loss was associated with subsequent achievement of ≥20% cumulative weight loss among patients who had not reached this threshold, but did not independently predict additional weight loss during months 6–12. Early response should not be used as a treatment-stopping rule.

## CRediT authorship contribution statement

Nikolaos Angelopoulos: Conceptualization, Methodology, Investigation, Data curation, Formal analysis, Writing – original draft, Writing – review & editing, Project administration, Supervision. Evangelos Fousteris: Investigation, Data curation, Writing – review & editing. Dimitra Zianni: Investigation, Data curation. Ioannis Androulakis: Investigation, Data curation. Rodis Paparodis: Conceptualization, Investigation, Data curation, Writing – review & editing. Dimos Florakis: Investigation, Data curation. Andreas Rizoulis: Investigation, Data curation. Ilias Perogamvros: Investigation, Data curation. Ploutarchos Tzoulis: Investigation, Data curation, Writing – review & editing. Dimitrios Askitis: Investigation, Data curation. Georgia Argyrakopoulou: Investigation, Data curation. Evdoxia Gitsi: Investigation, Data curation. Sarantis Livadas: Investigation, Data curation. Zadalla Mouslech: Investigation, Data curation. Valentina Petkova: Investigation, Data curation. Alexander Kokkinos: Writing – original draft, Writing – review & editing, Supervision. Vaia Lambadiari: Investigation, Data curation, Writing – review & editing.

## Authorship statement

All authors meet the journal's authorship criteria, have made sub stantial contributions to the study, and take responsibility for the integrity of the work.

## Ethics declaration

This study was conducted in accordance with the principles of the Declaration of Helsinki and was approved by the Ethics Committee of the Hellenic Endocrine Network (Protocol No. N2024/0121316). The requirement for informed consent was waived by the Ethics Committee because of the fully anonymized and retrospective nature of the study.

## Data sharing statement

The datasets generated and analyzed during the current study are available from the corresponding author on reasonable request.

## Declaration of generative AI and AI-assisted technologies in the manuscript preparation process

AI tools were used solely for limited language support during manuscript preparation. They were not used in the study design, data collection or analysis, interpretation of findings, or scientific decision-making. All authors reviewed and approved the final manuscript.

## Funding

This research received no specific grant from any funding agency in the public, commercial, or not-for-profit sectors.

## Declaration of competing interest

The authors declare the following financial interests/personal relationships which may be considered as potential competing interests: Angelopoulos Nikolaos reports a relationship with Pharmaserve - Lilly that includes: speaking and lecture fees. Sarantis Livadas reports a relationship with Pharmaserve - Lilly that includes: speaking and lecture fees. Evangelos Fousteris reports a relationship with Pharmaserve - Lilly that includes: consulting or advisory and speaking and lecture fees. Georgia Argyrakopoulou reports a relationship with Pharmaserve - Lilly that includes: speaking and lecture fees. Alexandros Kokkinos reports a relationship with Pharmaserve - Lilly that includes: consulting or advisory, funding grants, and speaking and lecture fees. Alexandros Kokkinos reports a relationship with Novo Nordisk Inc that includes: consulting or advisory, funding grants, and speaking and lecture fees. Vaia Lambadiari reports a relationship with Pharmaserve - Lilly that includes: consulting or advisory, funding grants, and speaking and lecture fees. Vaia Lambadiari reports a relationship with Novo Nordisk Inc that includes: consulting or advisory, funding grants, and speaking and lecture fees. If there are other authors, they declare that they have no known competing financial interests or personal relationships that could have appeared to influence the work reported in this paper.
