# Supplementary material for: 12-month weight-loss trajectories and predictors of response to tirzepatide under individualized dosing: A multicenter real-world study
Source: Obes Pillars. 2026 Oct 12;20:100344. doi: 10.1016/j.obpill.2026.100344 (PMC13683030; doi:10.1016/j.obpill.2026.100344)
Supplement: Supplementary file 1 [file mmc1.docx]

**Supplementary Table S1.**

**Distribution of study participants across the 13 participating clinical practices**

| **Clinical practice** | **Participants (n)** | **Percentage (%)** |
| --- | --- | --- |
| Center 01 | 56 | 22.5 |
| Center 02 | 31 | 12.4 |
| Center 03 | 29 | 11.6 |
| Center 04 | 22 | 8.8 |
| Center 05 | 21 | 8.4 |
| Center 06 | 19 | 7.6 |
| Center 07 | 13 | 5.2 |
| Center 08 | 12 | 4.8 |
| Center 09 | 11 | 4.4 |
| Center 10 | 11 | 4.4 |
| Center 11 | 10 | 4.0 |
| Center 12 | 8 | 3.2 |
| Center 13 | 6 | 2.4 |
| Total | 249 | 100.0 |

*Data are presented as number of participants (n) and percentage of the total study population (N=249). Participating clinical practices were anonymized and assigned numerical identifiers in descending order of sample size. Percentages may not sum to exactly 100% because of rounding.*

**Supplementary Table S2. Baseline characteristics and 6-month weight-loss response according to availability of 12-month weight measurements.**

| **Characteristic** | **12-month weight available (n=180)** | **12-month weight unavailable (n=69)** | **P value** |
| --- | --- | --- | --- |
| Age | 48.9 ± 10.7 (n=180) | 49.9 ± 11.0 (n=69) | 0.506 |
| Baseline weight (kg) | 117.0 ± 30.3 (n=180) | 104.7 ± 20.7 (n=69) | <0.001 |
| Baseline BMI (kg/m²) | 40.0 ± 8.5 (n=180) | 37.2 ± 6.8 (n=69) | 0.008 |
| 6-month weight loss (%) | 15.0 ± 6.5 (n=179) | 13.3 ± 6.5 (n=44) | 0.133 |
| Female | 111/180 (61.7%) | 52/69 (75.4%) | 0.053 |
| T2DM | 24/180 (13.3%) | 10/69 (14.5%) | 0.838 |
| Prior GLP-1 use | 58/180 (32.2%) | 25/69 (36.2%) | 0.552 |
| Statin use | 54/180 (30.0%) | 29/69 (42.0%) | 0.098 |

*Values are mean ± SD or n/N (%). Continuous variables were compared using Welch’s t-test and categorical variables using Fisher’s exact test. Six-month weight-loss data were available for 179 and 44 participants, respectively.*
